# 

**Published:** 1885-01

**Authors:** 


### COLLABORATORS.

CHICAGO:

PROFESSOR J. A. ALLEN, M. D., LL. D.
PROFESSOR E. ANDREWS, M. D., LL. D.
PROFESSOR W. H. BYFORD, A. M., M. D.
PROFESSOR O. C. DEWOLF, A. M., M. D.
PROFESSOR E. C. DUDLEY, A. B., M. D.
PROFESSOR J. H. ETHERIDGE, A. M., M. D.
PROFESSOR C. FENGER, M. D.
PROFESSOR J. N. HYDE, A. M., M. D.
PROFESSOR H. A. JOHNSON, M. D., LL. D.
CHARLES GILMAN SMITH, A. M., M.D.
J. F. TODD, M. D.

MILWAUKEE, WISCONSIN:

N. SENN, M, D.

WASHINGTON, D. C:

S. O. RICHEY, M. D.

UNITED STATES NAVY:

MEDICAL DIRECTOR J. M. BROWNE, M. D.

### CONTRIBUTORS OF ORIGINAL ARTICLES.

WILLIAM H. BYFORD.
HENRY J. REYNOLDS.
FRANK CARY.
C. C. WEBSTER.
D. A. K. STEELE.
JOSEPH ZEISLER.
FRANKLIN H. MARTIN.
G. H. CHAPMAN.
JAMES NEVINS HYDE.
CHRISTIAN FENGER.
HENRY T. BYFORD.
E. J. DOERINIG.
J. SUYDAM KNOX.
CHARLES CALDWELL.
E. L. HOLMES.
C. W. PURDY
W. J. WEBB.
H. GRADLE.
F. E. WAXHAM.
BOERNE BETTMAN.

